# The future of conferences

**DOI:** 10.1242/dev.200438

**Published:** 2022-01-04

**Authors:** Sally Lowell, Alistair Downie, Holly Shiels, Kate Storey

**Affiliations:** 1 Centre for Regenerative Medicine, Institute for Stem Cell Research, School of Biological Sciences, University of Edinburgh, 5 Little France Drive, Edinburgh EH16 4UU, UK; 2 Head of Information Technology, The Gurdon Institute, University of Cambridge, Tennis Court Road, Cambridge CB2 1QN, UK; 3 Faculty of Biology, Medicine and Health, Core Technology Facility, University of Manchester, 46 Grafton Street, Manchester M13 9NT, UK; 4 Division of Cell & Developmental Biology, School of Life Sciences, University of Dundee, Dow Street, Dundee DD1 5EH, UK

## The need for change

One of the many great things about being a developmental biologist is our strong sense of community. Conferences of all shapes and sizes reinforce these bonds between us and yet, even before the pandemic, scientists were becoming increasingly reluctant to travel to conferences due to concerns about climate change:

‘ Don’t think I will travel to conferences as delegate again. Cutting down as speaker a lot.’

Prof. Michael Stumpf (@theosysbio). Twitter; December 2019.

Younger scientists in particular expressed concerns about the environmental impact of their travel, but also worried about how not travelling might affect their career:

‘I know many senior scientists who are reducing attendance, but for someone like me without that recognition, how do I get my name out there without physically attending? Would love other options!’

Dr Louise Stephen (@ciliaNcilia). Twitter; December 2019.

Concerns about the changing climate were not the only problem. Traditional conference models also tend to exclude people with caring duties and those who can’t travel due to lack of funding or difficulties obtaining visas (Sarabipour, 2020; Sarabipour et al., 2021). As we approached 2020, it became increasingly clear that things needed to change.

Then came the pandemic. We got used to connecting with people, sharing and discussing data, and even socialising and networking, all through a computer screen. For many people, the online format has brought benefits even beyond the improvements in sustainability and accessibility:

‘For the first time, I actually mustered up the courage to ask a question in a talk at ISSCR! The chat is helpful for introverts like me.’

Mary Heather Celine Florido (@KiwiFlorido). Twitter; June 2020.

There are, however, downsides to the new online world: awkward online poster sessions, unsatisfactory online networking and, at least for some, a sense that they were becoming disconnected from their scientific community. As we emerge into 2022, things are changing again and our new normal is characterised by hybrid conferences, for example the upcoming joint Spring Meeting of the British Societies for Developmental Biology and Cell Biology, which will adopt a hybrid format for the first time. This, in some ways, gives us the best of both worlds, but also shares some of the same problems as fully remote conferences. Hybrid meetings create an additional risk that remote attendees may feel like second-class citizens (Parncutt et al., 2021). Getting the balance right between in-person and online also creates uncertainty for organisers: will there be enough in-person attendees for the meeting to work successfully? If a high proportion of delegates decide to switch from in-person to online at short notice, then will the online provision be sufficient to sustain the conference?

All of this leaves us with a problem. We urgently need to change the way we run our conferences, but many of us worry we might be losing too much if we limit ourselves to the existing remote and hybrid conference models.

## The need for experiments

Scientists do experiments. When things are not working optimally we try again, we fail again, we fail better. Eventually, we improve things. Yet, before the pandemic the traditional conference model remained, well… traditional, with little innovation. The pandemic has forced us into new ways of coming together to share our science: if we are not completely happy with them, should this drive us back to our old ways? We propose that we should instead embark upon a period of experimentation with conference models.

One particularly promising experimental approach is represented by ‘spatially distributed’ international conferences (Abbott, 2020; Parncutt et al., 2021). These bring people together in-person while limiting long-distance travel by organising into a number of hubs, linked together through streaming technologies. Such multi-hub conferences have already proved successful in other fields (Parncutt et al., 2021). This model could work particularly well for fields such as developmental biology, where many countries already have thriving national societies or regional meetings that could form local hubs within international spatially distributed conferences.

We’d suggest that some travel between hubs should be encouraged. In particular, cohorts of younger delegates might be keen to travel beyond their own country to expand their networks, and a few well-established international scientists might also switch places between hubs. This would enrich the experience for those who wish to (or need to) stay closer to home, while still greatly reducing overall air travel compared with a typical large international meeting.

By breaking large meetings up into multiple smaller hubs, we could encourage a more retreat-like atmosphere, perhaps with core workshop-like sessions on specific topics, giving opportunities for early career researchers (ECRs) to get focused feedback. These smaller sessions may also provide an ideal setting for productive discussions with remote contributors to the conference. This format would be a refreshing contrast to the typical traditional large international conference, which can be intimidating for younger researchers or those new to the field. So many of us have had the experience of arriving hopefully at these huge meetings, excited about meeting new colleagues, only to leave disappointed.

Multi-hub conferences could therefore solve a number of problems, but they could also bring new problems of their own. The organisation of these meetings could be complicated and potentially expensive. We sorely need reliable sources of information, particularly on logistics and technologies, in order to bring this promising new conference model within the grasp of the typical academic organiser.

Scientific meetings can be distributed in time as well as in space. A number of excellent field-specific online seminar series (webinars) have sprung up during the COVID-19 pandemic, with one particularly successful example being the Virtual Gastrulation Zoom Talks (VGZT; Twitter: @VGZT2020_21; see https://thenode.biologists.com/webinar-list/ for more examples). Such ventures can be more than just a series of talks; VGZT has fostered ongoing lively discussion and networking through asynchronous discussions on Slack between participants, many of whom return week after week. Other webinars have a broader scope. Development’s own ‘Development presents…’ series showcases the recently published or unpublished work of ECRs with a monthly menu of three short talks, often around a theme, followed by online small group discussions and socialising using Remo software (https://thenode.biologists.com/devpres/). Overall, webinars have been extremely helpful for keeping us in touch with each other in recent times, although most people would think of them as a complement to, rather than a replacement for, conferences. Plus, of course, they still lack the personal face-to-face interactions that so many people have missed.

## The need for creativity

Scientists are creative people. Ideas must be out there that can overcome or bypass some of the problems discussed above. For example, how about visiting conferences around the world in the form of a holograph or avatar (Le et al., 2020) (Box 1)? Or, maybe the traditional scientific talk, which can be delivered successfully online, could be completely separated from the free-flowing discussion of science, which tends to happen best in person. To take this second idea to its extreme, we could imagine one single large event each year that brings together all developmental biologists (or – let’s think big – all biologists) into an Expo-like festival with a varied menu of scientific and social activities. These activities could include lightning talks, poster sessions and focused workshops to provide a platform for ECRs and kick-start conversations, but would not include standard conference talks. Instead, we’d have watched these talks in our favourite online seminar series over the preceding year. Could the ‘Biology Expo’ give us all the benefits of traditional conferences at the bargain cost – in both money and carbon – of no more than one journey each year?

Smaller, but equally creative, ideas pop up from time to time in blogs and on social media. For example, how about the ‘poster bot’, an interactive mobile video-conferencing interface that could be based on ‘Meeting Owl’ technology (https://sustainability.biologists.com/blog/meeting-Owls-and-Poster-Bots-2/). We imagine the poster bot might resemble a friendly species of Dalek that has somehow acquired a surprisingly broad grasp of developmental biology. Your poster bot would roam around a poster session – or even a bar or coffee room – and allow you to join conversations from the comfort of your own sofa at home.

These ideas are not presented here as ready-to-use solutions. Rather, our point is that we don’t have all the solutions right now, so we must all think outside the box and share our suggestions. From this melting pot of ideas new solutions will emerge. But what is the mechanism for capturing and developing new ideas?

## The need for resources

Experiments are always a step into the unknown. A great conference should spark transformative ideas and collaborations and can be a life-changing experience, particularly for early career researchers. No conference organiser wants to risk all this by adopting an experimental conference design that might fall flat. There is also a financial risk in moving away from established conference models. New technology costs money, and delegates may be unwilling to pay the true cost for remote access to a conference. It can also be difficult for organisers to predict how many delegates will register for a meeting that adopts an unfamiliar format.

We propose that we need three types of resource to limit these risks:

1. **Information** about what worked and what failed for others.
2. **Discussion** of new ideas.
3. **Funding** to pay for new technologies and to mitigate financial risk.

Some excellent sources of information are already available online. These include: The Company of Biologists Sustainable Conferencing initiative (https://sustainability.biologists.com); the ‘Flying Less’ initiative to reduce academia’s carbon footprint (https://sites.tufts.edu/flyingless/multi-site-low-carbon-conferences-mulch/); resources from EMBL to help design virtual conferences (https://blogs.embl.org/events/2020/04/09/how-to-turn-your-conference-into-a-virtual-event/); ‘the Green Room’, a discussion forum for event planners to chat about sustainable events (https://forum.legacy-events.com/); and the YouTube channel of the Max Perutz Labs Climate Group (https://www.youtube.com/channel/UCwxfXV1iY2ROr6hf3zDH5JQ).

In particular, The Company of Biologists Sustainable Conferencing website aims to be a one-stop shop, collecting together information sources, summarising new developments through a series of blogs, and allowing anyone to feed in their own ideas through a discussion board or on Twitter (follow @COB_Sustainable and use #Sustainable_Conferencing to share your own thoughts). Importantly, they also provide grants to support new conference models and ask grantees to report back on what worked well and what worked less well in their particular experiment (Box 2).

## Summing up: the future of conferences

There will be no single ‘one size fits all’ solution to creating conferences fit for our new future. Pre-pandemic, most conferences tended to adopt a standard structure with very little evolution or experimentation. In the future, we envisage a ‘mix and match’ model based on a library of solutions that can be adapted to the particular needs of each conference.

This is not just a problem to be solved by conference organisers: everyone has a part to play. As Steve Royle puts it:

‘We can choose which meetings we attend. As a community, we can support meetings that are organised by organisations whose mission is to support science.’

(Steve Royle; https://quantixed.org/2021/10/29/pleased-to-Meet-Me-Returning-to-in-Person-Meetings/)

As he points out elsewhere in his article, we can also choose meetings organised with sustainability and accessibility in mind (see also Hamant et al., 2019; Sarabipour et al., 2021). In addition, we can all help by generating and discussing creative ideas and suggestions. What do *you* think would make conferences better for *you*? If you have an idea, we encourage you to discuss it with your colleagues and with the wider developmental biology community – who knows where it might lead. Let’s work together for a better future for conferences.

## Box 1 From Altspace to the Metaverse: a holographic space between traditional and hybrid?

Holograms provide a way to ‘be in the room’ without being there and have captured imaginations and conveyed important messages ever since Princess Leia appeared as a hologram in *Star Wars*. Their use for major events, such as concerts (think 2022 ABBA tour), are making them more mainstream and there are companies (e.g. ARHT Media, Spatial) currently making 3D holographic displays for workplace meetings that could transform scientific events. Imagine each hub at a multi-hub conference with the ability to ‘beam’ their speakers to plenary stages across space and time so that those physically there listening feel connected to the virtual holographic speaker.

Alternatively, we can give up the physical and go completely virtual. Virtual (VR) and augmented (AR) reality have been connecting people (well, their avatars) in virtual space for decades, but adopting their use for scientific meetings, workshops and conferences could provide additional ways for scientists to connect without being physically together.

Microsoft already offers AltspaceVR (https://altvr.com/), where users across AR/VR space can interact and attend or host meetings, events and workshops. The utility of the platform for scientists is growing as AltspaceVR aligns with ‘Mesh’, Microsoft’s mixed reality platform, providing VR avatars, holographic avatars and 2D PC users a space to come together ‘with a feeling of presence’. Facebook’s Horizon is a similar idea whereby meetings and events occur in the ‘Metaverse’ (https://www.wired.com/story/what-is-the-metaverse/). Online meetings and events in virtual space, or in real space with holographic attendees, are already happening. As mixed-reality technology improves, the utility of these environments for scientific conferences will grow.
